# UTR extension and alternate polyadenylation in neuroplasticity: an emerging paradigm?

**DOI:** 10.1186/1471-2105-15-S10-P11

**Published:** 2014-09-29

**Authors:** Benjamin J Harrison, Robert M Flight, Abdallah M Eteleeb, Eric C Rouchka, Jeffrey C Petruska

**Affiliations:** 1 Department of Anatomical Sciences and Neurobiology, University of Louisville, Louisville, KY 40202, USA; 2 Department of Cellular and Molecular Biochemistry, University of Kentucky, Lexington, KY, 40508, USA; 3 Department of Computer Engineering and Computer Science, University of Louisville, Louisville, KY 40292, USA; 4 Department of Neurological Surgery, University of Louisville, Louisville, KY 40202, USA; 5 Kentucky Spinal Cord Injury Research Center, University of Louisville, Louisville, KY 40202, USA

## Background

The 3’-untranslated region (3’UTR) of mRNA transcripts contributes to cell-type specific or developmental-stage specific regulation of gene functions by modifying cellular localization, stability and/or translational efficiency of transcripts.

## Materials and methods

Using RNA-seq to profile transcripts from neural tissue undergoing axonal plasticity, we detected approximately 1000 previously uncharacterized 3’UTR sequences, of which more than 100 are highly regulated when plasticity is induced.

## Results

Computational analyses of the novel UTR sequences, focusing on RNA-binding protein (RNAbp) interaction motifs revealed strongly over-represented RNAbps with known roles in nervous system pathologies. We consider the implications of 3’UTR transcript extension and protein interaction in the context of axonal plasticity and the consequences of mis-regulation of this process during neurological disease.

